# Cyclic Peptides for the Treatment of Cancers: A Review

**DOI:** 10.3390/molecules27144428

**Published:** 2022-07-11

**Authors:** Dalifa Ramadhani, Rani Maharani, Amirah Mohd Gazzali, Muchtaridi Muchtaridi

**Affiliations:** 1 Department of Pharmaceutical Analysis and Medicinal Chemistry, Faculty of Pharmacy, Universitas Padjadjaran, Jl. Raya Bandung-Sumedang KM 21, Sumedang 45363, Indonesia; dalifa20001@mail.unpad.ac.id; 2 Department of Chemistry, Faculty of Mathematics and Natural Sciences, Universitas Padjadjaran, Jatinangor 45363, Indonesia; r.maharani@unpad.ac.id; 3 School of Pharmaceutical Sciences, Universiti Sains Malaysia, USM, Penang 11800, Malaysia; amirahmg@usm.my

**Keywords:** anticancer, cyclic peptide, mechanism of anticancer, in vitro, in vivo

## Abstract

Cyclic peptides have been widely reported to have therapeutic abilities in the treatment of cancer. This has been proven through in vitro and in vivo studies against breast, lung, liver, colon, and prostate cancers, among others. The multitude of data available in the literature supports the potential of cyclic peptides as anticancer agents. This review summarizes the findings from previously reported studies and discusses the different cyclic peptide compounds, the sources, and their modes of action as anticancer agents. The prospects and future of cyclic peptides will also be described to give an overview on the direction of cyclic peptide development for clinical applications.

## 1. Introduction

Cancer is a non-communicable disease that has a high prevalence and causes mortality after cardiovascular and respiratory disorders [1]. It is caused by the abnormal growth of body cells that could metastasize to different organs from its origin. This disease is multifactorial, with factors such as population structure, lifestyle, and genetics being reported as among the contributing factors to its development and progression [2,3].

There are many approaches in cancer treatment currently available in clinical practice, with the most widely employed being surgical therapy, radiation therapy, and chemotherapy. However, many of these available treatment options lack selectivity, which causes side effects such as toxicity to the normal cells and damage to the surrounding tissues [4].

**Figure 1:**
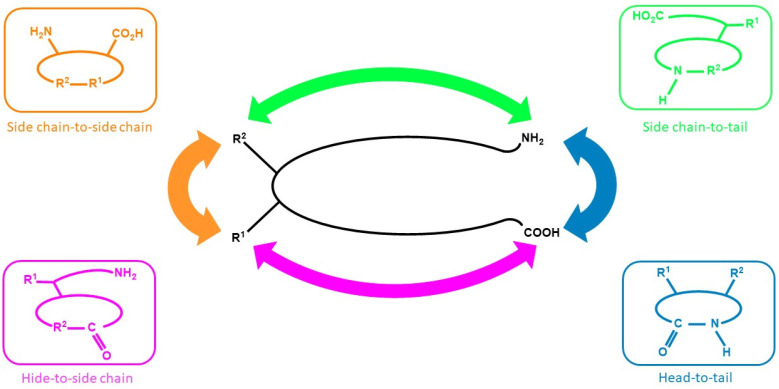
Cyclic peptides in general.

The ongoing research on new drug discovery and development against cancers has brought forward the potential of cyclic peptides as anticancer agents. Cyclic peptides, which are a type of polypeptide chain compound, contain a circular (cyclic) sequence of bonds (Figure 1) and they have been recognized for their medicinal properties [5,6,7]. The utilization of cyclic peptides as pharmaceuticals offers advantages because they have good binding affinity, target selectivity, and low toxicity, making them an attractive treatment modality [8]. In addition, cyclic peptides are not easily recognized by protease enzymes; thus, they have better stability than their linear counterpart [9], and they are also suitable to be used against extracellular targets [10]. Although some peptides are available from natural sources, most of the known peptides are encrypted in the structure of parent proteins and are mainly released via enzymatic processes [11].

Numerous studies have reported the anticancer activity of cyclic peptides. Previous reports in the literature have described the activity of cyclic peptides against breast cancer [12], lung cancer [13], and brain cancer [14], among others. This review summarizes the multitude of studies and data available on the application of cyclic peptides, specifically in breast, lung, colon, pancreas, liver, lymphoma, blood, and melanoma cancers. The study findings will be described, including the sources of cyclic peptides and their modes of action. In addition, the prospects and future of cyclic peptides as anticancer agents will also be described to provide an overview on the direction of cyclic peptide development for clinical applications.

## 2. Materials and Methods

The articles used in the preparation of this review were collected through a thorough search in journal indexing databases—Scopus, PubMed, and Google Scholar. The keywords used were “Cyclic peptide for breast cancer”, “Cyclic peptide for liver cancer”, “Cyclic peptide for colon cancer”, “Cyclic peptide for lung cancer”, “Cyclic peptide for pancreatic cancer”, “Cyclic peptide for prostate cancer”, “Cyclic peptide for lymphoma cancer”, “Cyclic peptide for blood cancer”, and “Cyclic peptide for melanoma cancer”.

**Figure 2:**
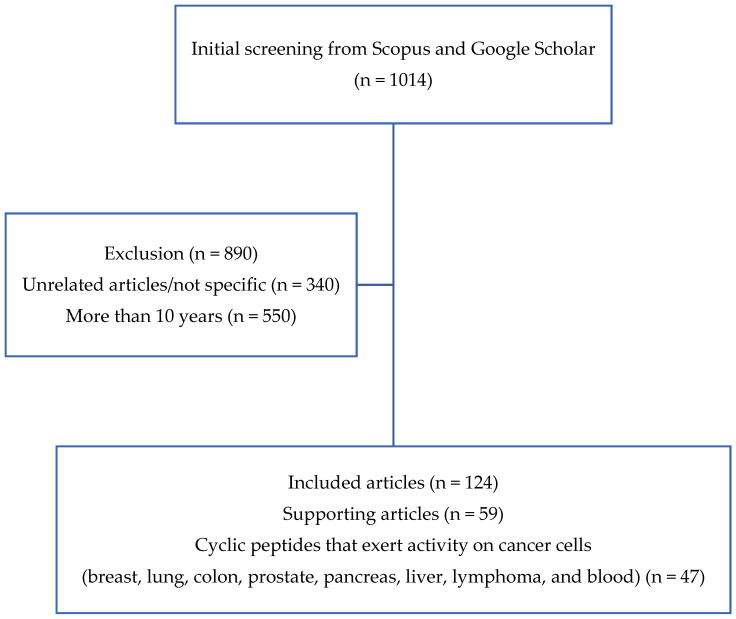
Literature search flowchart.

The inclusion criteria were as follows: (1) research articles, (2) cancer research, and (3) papers that described the utilization of cyclic peptides. Meanwhile, the exclusion criterion was articles that were published more than 25 years ago (before 1995). In the first stage of article collection, 1014 journal articles met the outlined criteria. However, after further examination, only 124 journal articles were chosen to be used for this review, which consisted of 75 articles on cyclic peptides with anticancer activity and 49 additional articles related to the mechanism and further supporting details. The article search flow is illustrated in Figure 2.

## 3. Structure and Source of Cyclic Peptide

### 3.1. Source and Physicochemical Characteristics of Cyclic Peptide

Cyclic peptides can be isolated from various sources, ranging from plants to marine biota [15,16,17]. In general, cyclic peptides consist of several amino acids in a specific sequence that are interconnected between the C-terminal and N-terminal, or between the head and the tail of the peptide sequence through an amide bond or other chemically stable bonds (Figure 1) [18,19]. Cyclic peptides have several favorable properties as compared with linear peptides. Geometrically, cyclic peptides can mimic active protein moieties to exert similar biological activities. Moreover, they can also increase the binding affinity of targeting motifs to certain receptor subtypes that are overexpressed by cancer cells, which results in better specificity towards the receptor subtype. In previously reported bioactivity studies, cyclic peptides containing cysteine residue were found to have a higher binding affinity for the targeted receptor than their linear counterpart [5,6,7].

In addition, the in vivo stability of linear peptides can be improved through transformation into cyclic peptides. The cyclization process has been shown to prolong the integrity of linear peptides, especially those with labile motifs present in the sequence. This is important, because peptide bonds are known to be susceptible to proteolytic degradation and could be spontaneously degraded under physiological conditions [20].

In cancer therapy and imaging, cyclic peptides have been shown to exert a higher binding affinity towards targeted receptors, in which they could displace endogenous proteins to disrupt the signaling cascades responsible for tumor growth. The cyclization of cell-penetrating peptides could improve their cell penetration ability, which may subsequently increase the therapeutic activity. In cancer imaging, radio-labeled cyclic peptides can function as radiotracers because they have a higher tumor uptake than healthy tissues when compared with linear radiolabel peptides. In addition, they do not require additional chelates to be used in radiotherapy, proving their versatility in radio-imaging applications [21].

### 3.2. Source of Cyclic Peptides

Cyclic peptides can be obtained from natural sources (plants, fungi, bacteria, sponges, algae, and mammals) [22], and they may also be chemically synthesized [23]. One example from plant isolates is RA-V, which was obtained from *Rubia cordifolia* L. and is known to have anticancer activity in breast cancer cells [24]. Another cyclic peptide, sansalvamide, was isolated from marine fungi of the genus *Fusarium* [25]. Isolation from bacteria has also been reported, and among the cyclic peptides obtained was a compound called wewakazole. This compound was isolated from two resources: (1) cyanobacterial *Lyngbya majuscula* off the coast of Papua New Guinea, and (2) *Moorea producens* in the Red Sea [26]. From the sponges, a cyclic peptide named stylopeptide 1 was reported to be isolated from the south and west Pacific Ocean, from sponges species called *Stylotella* sp. and *Phakellia costata* [27]. Cyclic peptides obtained from algae are galaxamide compounds isolated from the marine algae *Galaxaura filamentosa* [28]. In addition to these naturally derived cyclic peptides, many more have been chemically synthesized: one example is bacillistatin [29]. As shown Table 1, some of these compounds will be discussed in more detail in the following subsections.

#### Synthesis of Cyclic Peptides

**Figure 3:**
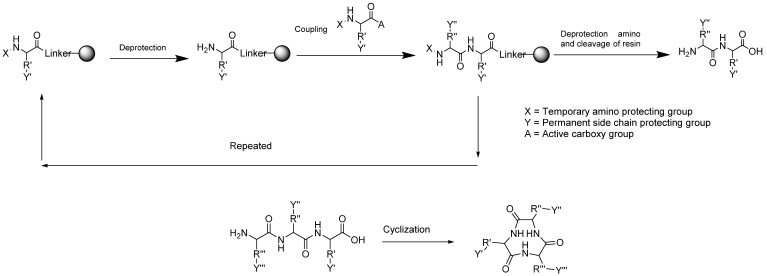
Synthesis of cyclic peptides through a combination of solid phase and liquid phase peptide synthesis strategies.

Strategies for the synthesis of cyclic peptide compounds often combine solid phase peptide synthesis and liquid phase peptide synthesis (Figure 3). In solid phase peptide synthesis, the strategy involves the selection of suitable resin, protective groups, and coupling reagents, whereas in liquid phase, it is necessary to select the cyclization center and coupling reagents. The solid phase approach has advantages of rapid product recovery, no repetition required, and simple product purification. However, this method is only suitable for small-scale production. The liquid phase approach, on the other hand, requires less reagent as compared with the solid phase strategy and produces good product yield, but a further purification process will be necessary. A combination approach is hence the best option to optimize product yield and purity [68,69].

## 4. Cyclic Peptides for Cancer

### 4.1. Cyclic Peptides for Breast Cancer

**Figure 4:**
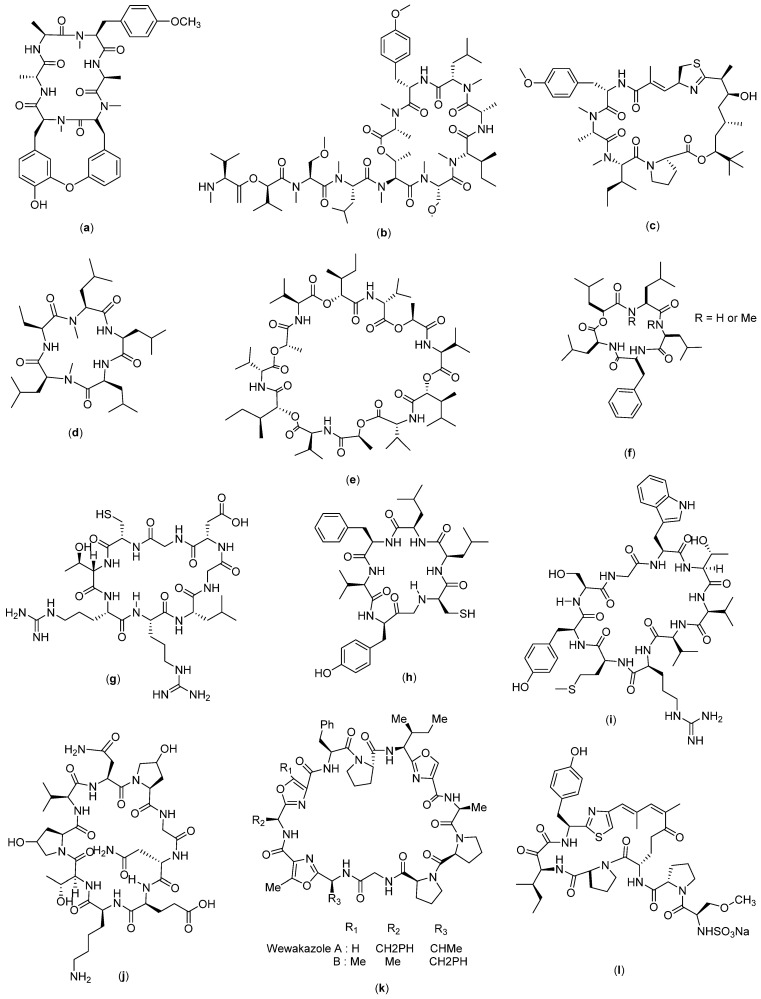
Structure of (**a**) RA-V; (**b**) Coibamide A; (**c**) Apratoxin A; (**d**) Galaxamide; (**e**) Bacillistatin 2; (**f**) Sansalvamide A; (**g**) Cyclosaplin; (**h**) Cyclo-CLLFVY; (**i**) Cyclo-SGWTVVRMY; (**j**) AFPep; (**k**) Wewakazole; and (**l**) Scleritodermin A.

There are several cyclic peptides that are active against breast cancer cells. RA-V (Figure 4a), which was isolated from *Rubia cordifolia* L., has anticancer activity in human breast cancer cells (MCF-7, MDA-MB-231 cells) and murine breast cancer cells 4T1 [24]. This compound triggers apoptosis in the mitochondrial pathway, which was observed as a loss of mitochondrial membrane potential, release of cytochrome c, and activation of the caspase cascade [70]. RA-V could inhibit the AKT-dependent phosphorylation of protein kinase 1 (PDK1) and 3-phosphoinositide in MCF-7 cells, in addition to disrupting the interaction between PDK1 and AKT in the cells, which contributes to its anticancer activity [24]. In addition, RA-V-induced apoptosis could be mediated by phosphatidylinositol 3-kinase inhibitor or may be attenuated by AKT over-expression in all three breast cancer cell types. These studies support the strong anti-tumor activity of RA-V against breast cancer.

Coibamide A (Figure 4b) and apratoxin A (Figure 4c) are also known to have anti-breast cancer activity [71]. Apratoxin A was isolated from marine *Moorea producens* and *Moorea bouillonii* [30,31], whereas coibamide A was isolated from the Panamanian marine cyanobacterium *Leptolyngbya* sp. [32]. Based on the study, it was found that coibamide A inhibited endothelial growth factor receptor (EGFR) expression and promoted EGFR degradation through a proteasome-dependent pathway [71]. In addition to its inhibitory activity on EGFR expression, coibamide A can interfere with cellular proteostasis and induce non-apoptotic cell death [72]. Coibamide analogues are known to be active against breast cancer xenografts MDA-MB-231 without inducing weight loss in tumor-bearing mice [73], and its effectiveness has been reported in the triple-negative breast cancer cells MDA-MB-468 [74]. In another study, this compound also showed a specific mechanism called the degradation of cotransin-induced Sec61 substrates [75], which has made it selective towards cancer cells [71]. Apratoxin analogues, on the other hand, were able to induce the lysosomal degradation of EGFR via chaperone-mediated autophagy [73] and kill the cells through direct blockage of the sec61 protein translocation channel [76].

Galaxamide (Figure 4d) is a cyclopentapeptide obtained from the isolation of marine algae *Galaxaura filamentosa* [33]. According to a reported study, galaxamide and its analogues exert anticancer activity in breast cancer cells through inducing cell apoptosis by disrupting the potential of mitochondrial membranes, producing ROS, and activating oxidative stress-mediated signaling in breast cancer cells. In addition, ROS generation will cause cell cycle arrest, which was reported to be the mechanism in MCF-7 cell apoptosis induced by galaxamide and its analogues [28].

Bacillistatin 2 (Figure 4e) is a series of synthetic cyclic peptides reported to have anticancer activity. In a related study, 35 analogues were synthesized and tested against breast cancer cells, from which 14 showed sufficient activity against the cells [29].

Sansalvamide (Figure 4f) is a cyclopentapeptide isolated from marine fungi of the genus *Fusarium* [25]. In a related study, 34 s-generation sansalvamide A (San A) derivatives were obtained. San A derivatives have shown anticancer properties towards colon, pancreatic, breast, prostate, and melanoma cancers. Its mechanism of action involves the inhibition of a key protein involved in tumor development (Hsp90) [77,78,79]. Hsp90 has a role in modulating the function of other proteins involved in apoptosis, cell growth, and angiogenesis, and it is one of the proteins that has been used as a target in conventional chemotherapy [80].

Cyclosaplin (4 g), which was purified from *Santalum album* L. somatic seed, was assayed against triple-negative human breast cancer cells (MDA-MB-231). This cyclic peptide compound showed significant antiproliferative activity with an IC_50_ of 2.06 µg/mL. In a mechanistic approach, apoptosis was observed in differential microscopic studies for peptide-treated MDA-MB-231 cells, which was further confirmed by mitochondrial membrane potential, DNA fragmentation assay, cell cycle analysis and caspase 3 activity. Co-localization studies revealed that the peptide sensitized MDA-MB-231 cells by possibly binding to EGFR and inducing apoptosis [34].

The cyclo-CLLFVY (Figure 4h) was obtained by tracing 3.2 million cyclic hexapeptide compounds using a genetically coded assessment platform (SICLOPPS library). This compound is a synthesized compound with a cyclic Cys-Leu-Leu-Phe-Val-Tyr amino acid composition which was proven to have activity against MCF-7. Its mechanism involves the inhibition of dimerization and transcriptional activity of HIF-1 by binding to the PAS-B domain of HIF-1α and the reduction in hypoxia-mediated response signaling [81].

Molecular design approaches have been widely used in designing new and active drugs. In order to design anticancer cyclic peptides, a modulator design was carried out by assessing the interactions of transcription factor proteins with cyclic peptide as a ligand to obtain an active ligand. Identification was carried out to screen a library of 64 million cyclic peptides on their ability to inhibit the NADH-dependent dimerization of the transcriptional repressor C-terminal binding protein (CtBP). CtBP dimers form the core of transcriptional complexes associated with the epigenetic regulation of several genes that control many characteristics of cancer cells, including proliferation, survival, and migration. The most potent inhibitor discovered from this study was the cyclonanopeptide Ser-Gly-Trp-Thr-Val-Val-Arg-Met-Tyr. Cyclo-SGWTVVRMY (Figure 4i) has been shown to interfere with CtBP dimerization in vitro. This compound was then used to establish the role of the NADH-dependent dimerization of CtBP in the regulation of mitotic processes in cancer cells [82].

AFPep (Figure 4j) is a cyclic compound with nine amino acids—Glu-Lys-Thr-O-Val-Asn-O-Gly-Asn—whereby O represents a hydroxyproline. This compound is an imitation of the AFP compound (fetoprotein) which has been shown to have anticancer activity in liver cancer [83] and gastric cancer [84]. This synthetic AFPep compound, on the other hand, was reported as having the ability to stop the growth of human breast cancer xenografts in SCID mice [85], whereas the prevention of recurrence was demonstrated against carcinogen-induced breast cancer in Sprague Dawley mice [86]. In addition, its low toxicity was also successfully demonstrated through the absence of interference with the rat estrus cycle, preservation of fertility, and absence of birth defects in the offspring [87].

Wewakazole (Figure 4k) could be obtained from two resources: (1) isolation from the cyanobacterial isolate *Lyngbya majuscula* off the coast of Papua New Guinea, and (2) isolation from *Moorea producens* in the Red Sea. The amino acid sequences of the two wewakazole species are slightly different, with the first type consisting of Gly-Pro-Pro-Ala-Oxz-Ile-Pro-Phe-Ile-Pro-Fall, whereas Gly-Pro-Pro-Ala-Oxz-Mele-Oxz-IleP-Phe forms the sequence of the second type of wewakazole, which is also known as wewakazole B. Wewakazole B is known to have toxicity on several cancer cell lines including MCF7 and H460 (lung cancer cell lines) with IC_50_ values of 0.58 µM and 1.0 µM, respectively. For wewakazole, on the other hand, no data are available on its anticancer bioactivity [26].

Scleritodermin A (Figure 4l) is a cyclopeptide compound obtained from the lithistid sponge *Scleritoderma nodosum*; its structure consists of L-proline, L-serine, and keto-allo-isoleucine units with a thiazole moiety (ACT) and O-methyl-N-sulfate. The compound has demonstrated anticancer activity against breast carcinoma SKBR3 with an IC_50_ value of 0.67 µM. Its reported mechanism of action is via inhibition of the tubulin polymer and subsequent disruption of the microtubule function. It is known to be important in mitotic spindle functions; therefore, microtubules are the targets of several naturally derived compounds that are clinically used as anticancer drugs such as paclitaxel [35].

### 4.2. Cyclic Peptide for Lung Cancer

**Figure 5:**
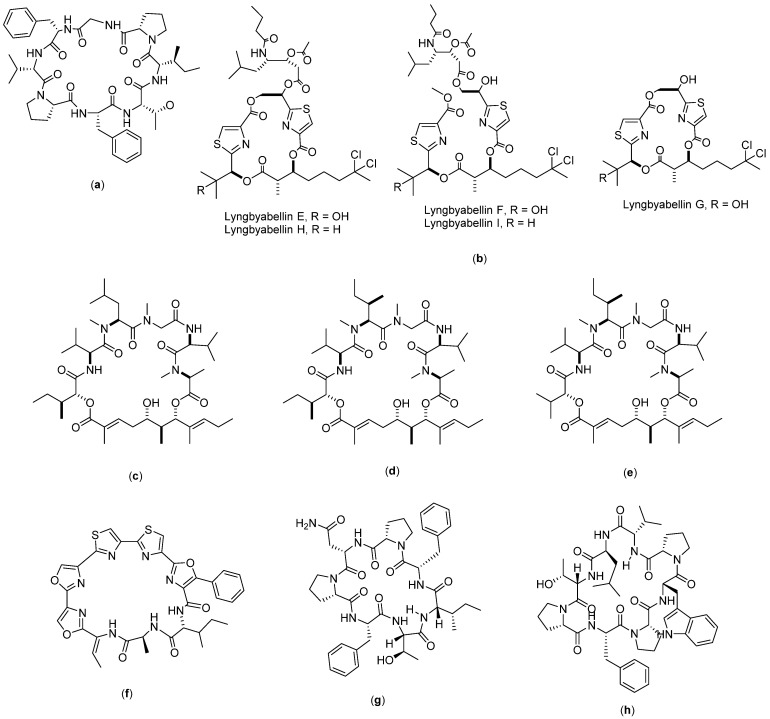
Structure of: (**a**) Psammosilenin; (**b**) Lyngbyabellin; (**c**) Aurilide A; (**d**) Aurilide B; (**e**) Aurilide C; (**f**) Urukthapelstatin A; (**g**) axinellin A; and (**h**) axinellin B.

Psammosilenin B (Figure 5a) is a cyclic peptide with a structure consisting of Pro-Gly-Phe-Val-Pro-Phe-Thr-Ile. It was isolated from *Psammosilene tunicoides* [88] and has shown significant anticancer activity against lung cancer cells (HOP-92). As reported by Shinde et al. (2015), this compound, when tested on A549/ATCC, HOP-62, HOP-92, NCI-H226, NCI-H23, NCI H322M, NCI-H460, and NCI-H522, exhibited an ability to inhibit these cells, particularly the HOP-92 cell line, with a 118.43% inhibition recorded [36].

Lyngbyabellin (Figure 5b) is a compound obtained from the isolation of the pantropical marine cyanobacterium, *Lyngbya majuscula*. The isolation produces nine lyngbyabellin compounds (lyngbyabellin A-I), from which five of them (lyngbyabellins E-I) have demonstrated cytotoxicity to human lung tumor NCI-H460 with LC_50_ values between 0.2 and 4.8 µM [89]. The mode of action was found to be through the induction of apoptosis through the impaired cytokinesis of cellular microfilament networks [37].

Aurilide (Figure 5c–e) is a depsipeptide compound consisting of three types, namely, A, B, and C. Aurilide A was obtained from the isolation of the marine biota *Dolabella auricularia* found in the Sea of Japan [90], whereas aurilides B and C were obtained from the marine biota strain cyanobacterium *Lyngbya majuscule* in Papua New Guinea [91]. Aurilide A consists of cyclo-[N(Me)Ala-Unk-Val-DN(Me)Leu-Sar-Val], whereas aurilide B is cyclo-[N(Me)Ala-Unk-Val-N(Me)aIle- Sar-Val], and aurilide C is cyclo-[N(Me)Ala-Unk-Val-N(Me)aIle-Sar-Val]. Aurilides B and C are known to have cytotoxicity on NCI H460 human lung cancer cells. Han et al. (2006) reported that aurilide B has a fourfold greater toxicity as compared with aurilide C. The IC_50_ value of aurilide B on NCI H460 was reported to be 0.01 µM, whereas for aurilide C, the IC_50_ recorded was 0.05 µM. The anticancer mechanism of aurilide is known to be through the induction of apoptosis by interfering with mitochondrial morphogenesis. Aurilide is also known to be enriched with prohibitin 1 (PHB1), a membrane protein from mitochondria, which chemically interferes with proteolytic atrophy optic (OPA1), giving aurilide the ability to inhibit mitochondrial fragmentation and apoptosis [38].

Urukthapelstatin A (Figure 5f) is a peptide isolated from mycelia cultured from the sea, which then produces the bacterium *Thermoactinomycetaceae Mechercharimyces asporophorigenens* YM11-542. Its structure consists of cyclo [Ala-Unk-D-aIle]. The compound is known to have cytotoxic properties in the A549 cell lines with an IC_50_ value of 12 nM. In another study, the compound urukthapelstatin A also showed cytotoxicity in different cancer cell lines, including MCF-7, HCT-116, A549, DMS114, and NCI H460, with log GI_50_ values between −8.28 M and −8.46 M. The authors concluded that urukthapelstatin has strong anticancer activity in comparison to cytarabine and enocitabine, which are two common anticancer drugs used in clinical practice [39].

Axinellin (Figure 5g–h) is a cyclopeptide compound with two analogues—axinellin A and axinellin B. They can be isolated from the marine sponge *Axinella carteri*. Axinellin A has an amino acid sequence consisting of seven amino acids (Ile-Phe-Pro-Asn-Pro-Phe-Thr), whereas axinellin B has a sequence consisting of eight amino acids (Val-Leu-Thr-Pro-Phe-Pro-Trp-Pro). The sequence of the axinellin A cyclopeptide has been successfully synthesized, as reported in the literature [92]. In terms of anticancer activity, both axinellins A and B showed moderate in vitro activity against the human bronchopulmonary non-small-cell lung carcinoma (NSCLC-N6) cell lines with IC_50_ values of 3.0 and 7.3 µg/mL, respectively. The mechanism of actions for their anticancer activity has not yet been fully understood based on the currently available literature [40].

### 4.3. Cyclic Peptide for Colon Cancer

**Figure 6:**
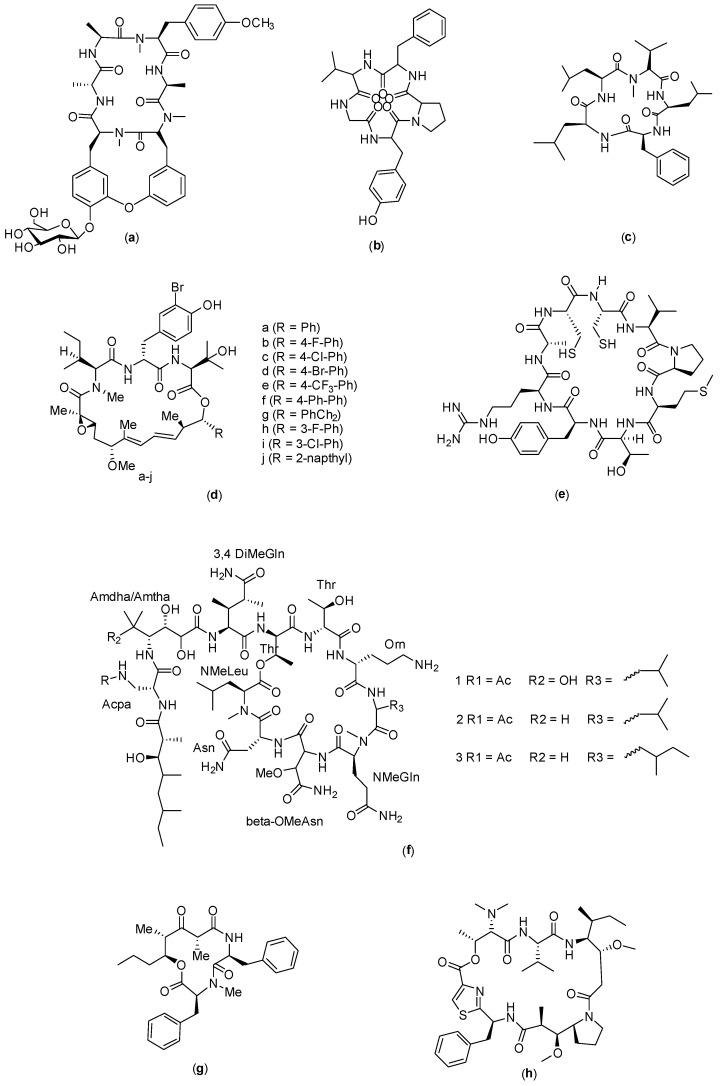
Structure of: (**a**) RA-XII; (**b**) Longicalcynin A; (**c**) Sansalvamide G; (**d**) Nannocystin (**e**) C25; (**f**) Theopapuamide; (**g**) Stereocalpin; and (**h**) Dol-10 cyclic.

RA-XII (Figure 6a) is a bicyclic hexapeptide compound isolated from *Rubia yunnanensis* Diels that has shown anticancer activity against colon cancer cells. This compound could exert an inhibitory effect on the growth of CRC 595 cells in vitro and in vivo. In a related study, the data obtained revealed that RA-XII-mediated cell death did not happen via apoptosis, but rather through the inhibition of protective autophagy in SW620 and HT29 cells. In addition, it was shown that RA-XII suppresses the initial stages of autophagy by activating the Akt–mTOR pathway and inhibiting autophagosome formation. Furthermore, RA-XII could also increase the sensitivity of CRC 595 cells towards bortezomib. This is a potentially useful synergistic effect in cancer treatment, and thus is a sign of a promising prospect for this molecule to be used in combination with other chemotherapeutic drugs against cancer cells [41].

Longicalcynin A (Figure 6b) is a cyclopeptide originally isolated from *Dianthus superbus* [52]; the peptide with its analogues were subsequently synthesized, as reported by Ahmad Waqar et al. (2013) [42]. The cytotoxicity of the synthesized compounds was evaluated to obtain information on the structure–activity relationship of the cyclopeptides, which could lead to the discovery of new, safer anticancer agents. The results obtained showed that two of the cyclopeptide analogues synthesized, with sequences of cyclo-(Thr-Val-Pro-Phe-Ala) and cyclo-(Phe-Ser-Pro-Phe-Ala), were cytotoxic to colon and liver cancer cells, with good safety profiles on fibroblast cells. These compounds were found to cause apoptosis on the targeted cells; it was observed that the integrity of the lysosomal membrane was lost following the treatment given to the cells [93].

Sansalvamide (Figure 6c) is a cyclopeptide originally isolated from fungi in the genus *Fusarium* [43]. Rodriguez et al. (2007) described the modification and synthesis of the sansalvamide analogues through which 14 derivatives were produced (sansalvamide A–N). In this study, sansalvamide G showed anticancer activity on HCT-116 colon cancer cells [94]. The original sansalvamide (A) also exhibited significant antitumor activity. In another study reported by Zhang and coworkers in 2014, a novel derivative of sansalvamide A called H-10 was synthesized, which exhibited an inhibitory effect on the proliferation of murine B16 melanoma cells and had a mechanism for inducing the apoptosis of B16 cells. Inhibition by H-10 in B16 cells was also found to occur in a concentration-dependent manner [95].

Nannocystin is a cyclic peptide compound that was isolated from the myxobacterial genus *Nannocystis* sp. [44] and subsequently modified to produce ten analogues (3a–j) (Figure 6d). The analogues were tested on several cancer cell lines, and the results showed that (3a,b,e,f,g,h) compounds demonstrated cytotoxic properties on several cancer cells, namely, HCT116 (colon), HepG2 (liver), PANC1 (pancreas), FHC (normal colorectal mucosa), and LO2 (normal hepatocytes) [96]. The mode of action for the compounds is through targeting eukaryotic translation elongation factor 1 A (eEF1A) [96,97].

Another study reported that the cyclic peptide compound C25 (Figure 6e), which has nine amino acid residues in the sequence Cys-Val-Pro-Met-Thr-Tyr-Arg-Ala-Cys, has cytotoxicity against colon cancer cells (CT26), as shown through an in vivo study conducted in rats [98]. It exerts its cytotoxicity by activating the CD8+ T cells, following which the concentration of FOXP3+ at the tumor site will be reduced [99]. In addition, this compound has a high affinity towards LAG-3, which will blockade the LAG-3 signaling pathway and produce the antitumor activity.

Theopapuamide (Figure 6f) is a compound isolated from the sea sponge *Siliquariaspongia mirabilis.* Theopapuamides A–D are undecapeptide compounds with an *N*-terminal fatty acid moiety containing two previously unreported amino acids, 3-acetamido-2-aminopropanoic acid and 4-amino-2,3-dihydroxy-5-methylhexano. Theopapuamide A–C compounds are known to exert cytotoxic activity on human colon cancer cells (HCT-16) with IC_50_ values between 2.1 and 4.0 µg/mL. In addition, this compound showed strong antifungal activity against wild-type and amphotericin-B-resistant *Candida albicans* strains at a load of 1−5 g/disk [45].

Stereocalpin (Figure 6g) is a cyclic depsipeptic obtained from the isolation of dry moss *Ramalina terebrata* with a structure consisting of cyclo-[Phe-N (Me) Phe-Unk]. Its cytotoxicity has been evaluated against several cancer cell lines, namely, the human colon carcinoma cell line (HT29), human skin carcinoma cell line (B16F10), and human liver carcinoma cell line (HepG2), which yielded IC_50_ values of 6.5 µM, 11.9 µM, and 13.4 µM, respectively. The mechanism of action has not yet been explored in depth, which is partly due to the lack of samples available for testing [46].

The cyclic analogue of the compound dollastatin 10 (Figure 6h) is the result of the cyclization reaction of the compound dolastatin (Dol-10). This compound is a pentapeptide obtained by isolation from mollusks *Dolabella auricularia* in the Indian Ocean; it contains unique asandam and has been reported to be effective in reducing apoptosis in cancer cells at nanomolar concentrations. It has the potential to treat lymphoma specifically, and reduce the lesions in the tumor. This cyclic analogue compound has shown cytotoxicity in HT-29 and L1210 cells with IC_50_ values of 58.5 nM and 110.8 nM, respectively. No preclinical or clinical data are available to date [47].

### 4.4. Cyclic Peptide for Prostate Cancer

**Figure 7:**
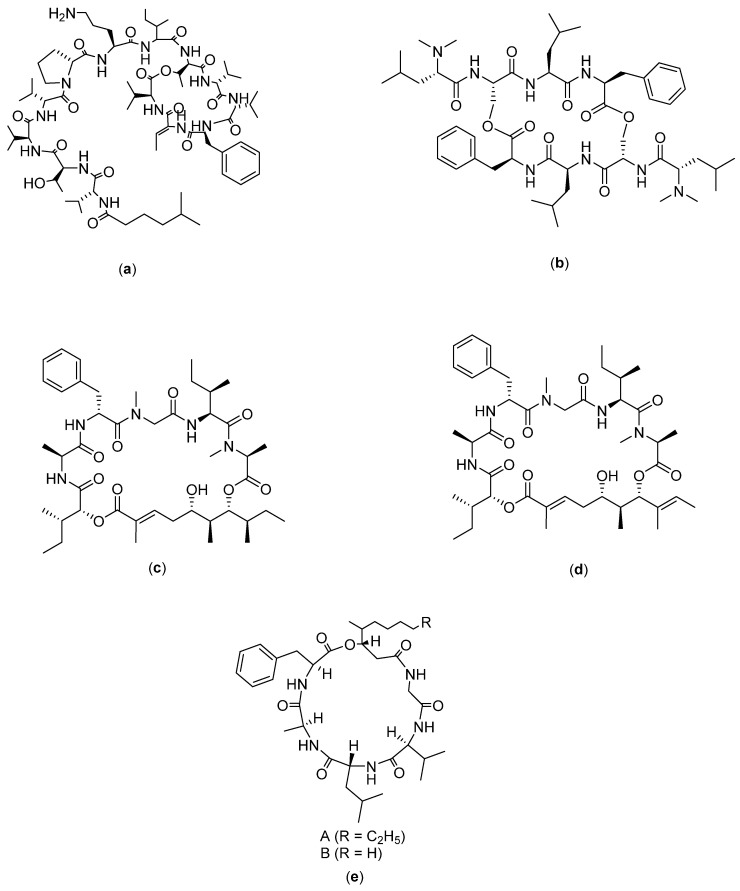
Structure of: (**a**) Kahalalide F; (**b**) IB-01212; (**c**) Lagunamides A; (**d**) Lagunamides B; and (**e**) Scopularides.

Kahalalide F (Figure 7a) is a partially cyclic depsipeptide group which is found in algae (*Bryopsis pennata*). Its main mechanism of action has not been identified; however, preliminary experimental results have provided some information regarding the physiological events that correlate with tumor cell death following treatment with the compound. In the NCI COMPARE analysis, kahalalide F did not show significant correlation with other standard chemotherapeutic agents, suggesting that it may have a unique means of achieving biological effectiveness. In an in vitro study reported by Suarez and co-workers, the prostate tumor cell lines PC-3 and DU-145 happened to be the most sensitive towards kahalalide F with an LC_50_ below 10 µM [100]. Neither protein nor nucleic acid synthesis were found to be inhibited in cultured cells by sublethal concentrations of kahalalide F. Topoisomerase (I or II) enzyme activity was also not affected; no DNA damage was specifically correlated with the exposure to kahalalide F. A cell cycle blockade at G0-G1 has been identified in a variety of cancer cells, including prostate (DU-145), cervical (HeLa), colon (HT-29), head and neck (HN30), and non-small-cell lung carcinoma (NSCLC; HOP62), all with IC_50_ values within the 1 µM range. This compound has been tested from preclinical to clinical settings for the treatment of prostate cancer [101]. Its mechanism of action is mostly through necrotizing cell processes that involve oncosis rather than apoptosis [102]. Thus far, there is only one published study on the chemical synthesis of kahalalide F [48].

IB-01212 (Figure 7b) is an isolate obtained from mycelium extract of *Clonostachys* sp. ESNA-A009. Compound IB-01212 is a cyclodepsipeptide compound which has structures as a cyclic dimer formed by two LN chains, N-Me2Leu-L-Ser-LN -MeLeu-LN-MePhe and bound by two esters formed between asPa the hydroxyl functional group of L-Ser. This compound has been tested in vitro on 14 different human tumor cell lines and it is most active against LN-caP (prostate cancer), SK-BR3 (breast cancer), HT29 (colon cancer), and HELA (cervical cancer), with GI_50_ (growth inhibition) values in the order of 10^−8^ M [49].

Lagunamides A and B (Figure 7c,d) are cyclic depsipeptides isolated from the filamentous marine cyanobacterium, *Lyngbya majuscula*, from Hantu Island, Singapore. Previous studies tested lagunamides A and B on several cancer cell lines, namely, BJ, BJ shp53, P388, A549, PC3, and SK-OV3, from which the cytotoxicity of lagunamide A was demonstrated on P388 (IC_50_: 6.4 µg/mL), A549 (IC_50_: 2.9 µg/mL), PC3 (IC_50_: 2.5 µg/mL), and SK-OV3 (IC_50_: 3.8 µg/mL), whereas lagunamide B is cytotoxic towards PC3, A549, and SK-OV3 cell lines [50].

### 4.5. Cyclic Peptide for Pancreatic Cancer

Scopularides A and B (Figure 7d) are two new cyclodepsipeptides, isolated from the fungi *Scopulariopsis brevicaulis*, which can be obtained from the marine sponge *Tethya aurantium*. Yu et al. (2008) reported that the compounds have activity against several tumor cell lines such as Panc89, Colo357, and HT29. At a concentration of 10 µg/mL, the two compounds showed similar inhibitory effects on Panc89 (pancreatic), Colo357 (colon), and HT29 (colon) cells. Treatment with scopularides A caused a reduction in pancreatic cancer cell line viability by 36%, colon cancer cell line (Colo357) by 42%, and HT29 by 37%, whereas scopularides B reduced the viability of pancreatic cancer cell line by 26%, colon cancer cell line (Colo357) by 49% and HT29 by 24%. The authors concluded that scopularides A is more toxic than scopularides B in the pancreatic cancer cell line (Panc89) [51].

### 4.6. Cyclic Peptide for Liver Cancer

**Figure 8:**
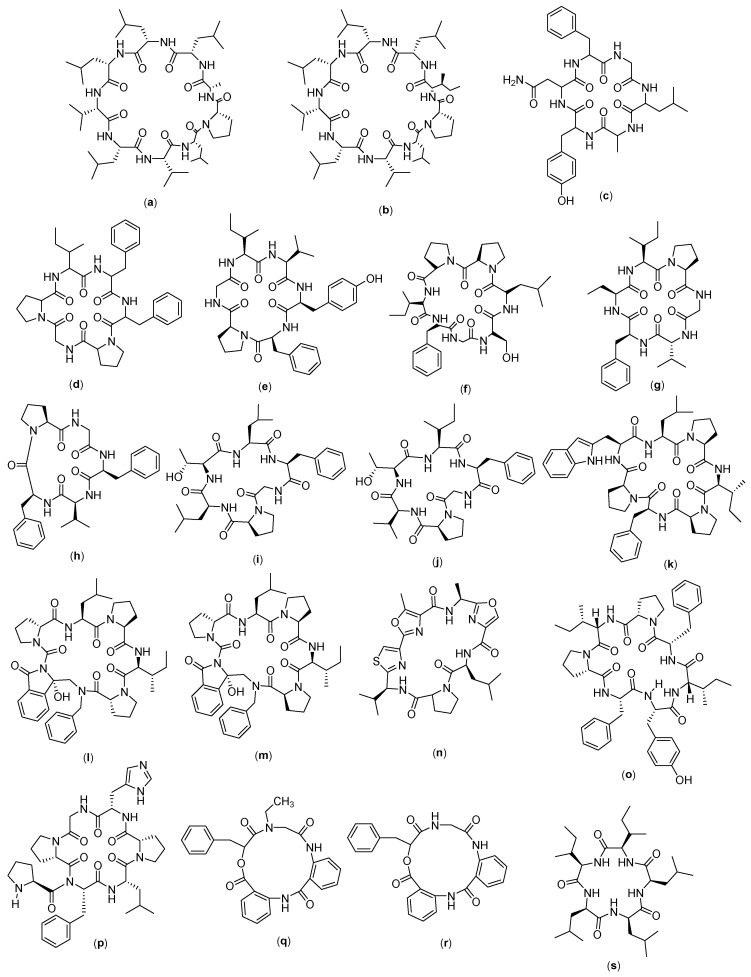
Structure of (**a**) Grifficyclocin B; (**b**) GG-8-6; (**c**) Dianthin A; (**d**) Dianthin B; (**e**) Dianthin C; (**f**) Dianthin D (**g**) Dianthin E; (**h**) Dianthin F; (**i**) Dianthin G; (**j**) Dianthin H; (**k**) Phakillestatin 6; (**l**) Analogs phakellistatin 6 (DLD-S); (**m**) Analogs phakellistatin 6 (LLD-S); (**n**) Leucamide; (**o**) Stylissatin A; (**p**) Stylissatin B; (**q**) Clavatustide A; (**r**) Clavatustide B; and (**s**) Clavatustide C.

Grifficyclocin B (Figure 8a) was originally isolated from the *Goniothalamus* (Annonaceae) plant, and it was then chemically modified to produce twelve analogues, as reported by Chen and co-workers [14]. The results of the bioassays showed that these synthetic cyclopeptides exhibited different levels of cytotoxicity against human liver cancer cells. Among them, GG-8-6 (Figure 8b) was the most active, with IC_50_ values of 6.38 µM and 12.22 µM against SMMC-7721 and HepG2, respectively. Further evaluation showed that the mechanism of action for GG-8-6 activity includes the induction of apoptosis and damages G2/M HCC cells. In addition, activation of the caspase pathway may be involved. Anti-tumor experiments in vivo demonstrated that GG-8-6 can significantly inhibit tumor growth in a mouse xenograft tumor model. At a dose of 40 mg/kg, the inhibition ratio was 67.9% without any weight loss [14].

Dianthin (Figure 8c–j) is an isolate compound obtained from a Chinese medicinal plant, called *Dianthus superbus.* Initially there were only two isolates; then, four cyclohexapeptides were successfully isolated. The compounds are referred to as dianthin A-F. Among the compounds, dianthin E has demonstrated cytotoxicity towards HepG2 liver cancer cells, but it has no activity against other cancer cell lines [103]. In another study, from *Dianthus superbus* var. longicalycinin, a cyclopentapeptide named longicalycinin A was isolated. The activity of longicalcynin A was evaluated towards liver (HepG2 and Hep3B), breast (MCF-7 and MDA-MB-231), and lung cancer (A-549) cell lines, with the most significant toxicity recorded on HepG2 [52], although this effect was less than that of dianthin E. Researchers have also demonstrated that the cytotoxicity of longicalycinin A in two cell lines from ascitic Dalton lymphoma (DLA) and ascitic Ehrlich carcinoma (EAC) is much higher than 5-FU, which was used as a positive control. Meanwhile, dianthin A was reported to have anticancer effects on DLA and EAC cell lines, comparable to 5-FU [104]. Longicalycinin A, which was chemically synthesized via a solid phase peptide synthesis strategy, was evaluated for its anticancer activity in EAC and DLA cell lines. It was found that longicalycinin A has cytotoxicity against Dalton’s lymphoma ascites (DLA) and Ehrlich’s ascites carcinoma (EAC) cell lines, with CTC_50_ values of 2.62 and 6.17 µM, respectively [93].

Phakillestatin 6 (Figure 8m) is a cyclic peptide compound which has a structure that shows interesting biological activity, due to the conformational stability provided by the proline residue. In their study, Zhao and coworkers synthesized phakellestatin and its analogues, which were then tested on HepG2, HeLa, and L929 cell lines. The results showed that the two analogues of phakillestatin 6 had IC_50_ values of 11.10 µg/mL (DLD-S) and 7.79 µg/mL (LLD-S) for HepG2. Furthermore, LDH activity assay and electron microscopy confirmed the occurrence of membrane disruption as the possible mechanism of action [53].

Leucamide (Figure 8n) is a peptide isolated from the Australian marine sponge *Leucetta mikrorapis*. This compound has a cyclo Leu-Oxz-Ala-MeOxz-Thiazole-Val-Pro structure, with activity against the HM02 cell line (GI_50_: 5.2 µg/mL), HepG2 (GI_50_: 5.9 µg/mL) and Huh7 (GI_50_: 5.1 µg/mL). However, to date, there are no reported data on the mechanism of action of this compound [54].

Stylissatin (Figure 8o–p) is a cyclic peptide compound that has seven amino acids and consists of four analogues, called stylissatin A–D. They can be isolated from the *Stylissa masse* sponge. Stylissatin A is known to have a cyclo-[Tyr1–Ile2–Phe3–Pro4–Ile5–Pro6–Phe7] structure and it inhibits the production of nitric oxide in murine macrophage cells RAW264 LPS with an IC_50_ value of 87 nM. The structure of stylissatin B is cyclo-[Pro1-Phe-Leu-Pro3-His-Gly-Pro2]. Stylissatin B is the most active as compared with other models of stylissatin. Stylissatin B has shown inhibitory activity on several cancer cell lines, which include HCT with 4.4 µM, HepG2 with 2.3 µM, BGC-823 with 10.6 µM, NCI-H1650 with 9.8 µM, A2780 with 3 µM, and MCF7 with 4.8 µM [55].

Clavatustide (Figure 8q–s) is a cyclic peptide compound isolated from the metabolites of mycelia cultivated with the hydrothermal fungus *Aspergillus clavatus*. Clavatustide consists of several types, namely, A, B, and C. Clavatustide A is a depsipeptide with a cyclo structure [2Abz-2Abz-D-OPhe-Sar], and clavatustide C has the structure of cyclo- (L-Ileu-L-Ile-L-Leu-L-Lue-L-Leu) cyclo [L-leucyl-L-leucyl-L-leucyl-(3xi)-L-isoleucyl- (3xi)-L-isoleucyl]/cyclo [Leu-Leu-Leu-xiIle-xiIle]. Clavatustides A and B have been synthesized [105]. Clavatustides A and B are known to have anticancer activity on the liver, with the A analogue having far better activity than the B analogue. This can be seen from the assay of these two compounds on several normal human hepatocyte cells (L02 cells) and all HCC (HepG2) cell lines, SMM-HCG22, in which the data obtained support the superior activity of the A analogue lower Max effect (30 µg/mL vs. 50 μg/mL, *p* < 0.05) and IC_50_ dose (15 µg/mL vs. 25 µg/mL, *p* < 0.05). These two compounds were further tested to determine their mechanism of action and showed that clavatustide A and B regulate the G1-S transition in liver cancer cell lines. Other findings looked at the role of clavatustide B in other cancer cell lines, including human pancreatic cancer (Panc-1), gastric cancer (MGC-803), colorectal cancer (SW-480), retinoblastoma (WERI-Rb-1), and prostate cancer (PC3). Clavatustide B is an effective anti-proliferative drug with a dose-enhancing effect on the whole blood cell with the control of it. The reduction in the number of viable cells became significant at 10–20 µg/mL [56].

### 4.7. Cyclic Peptide for Lymphoma Cancer

**Figure 9:**
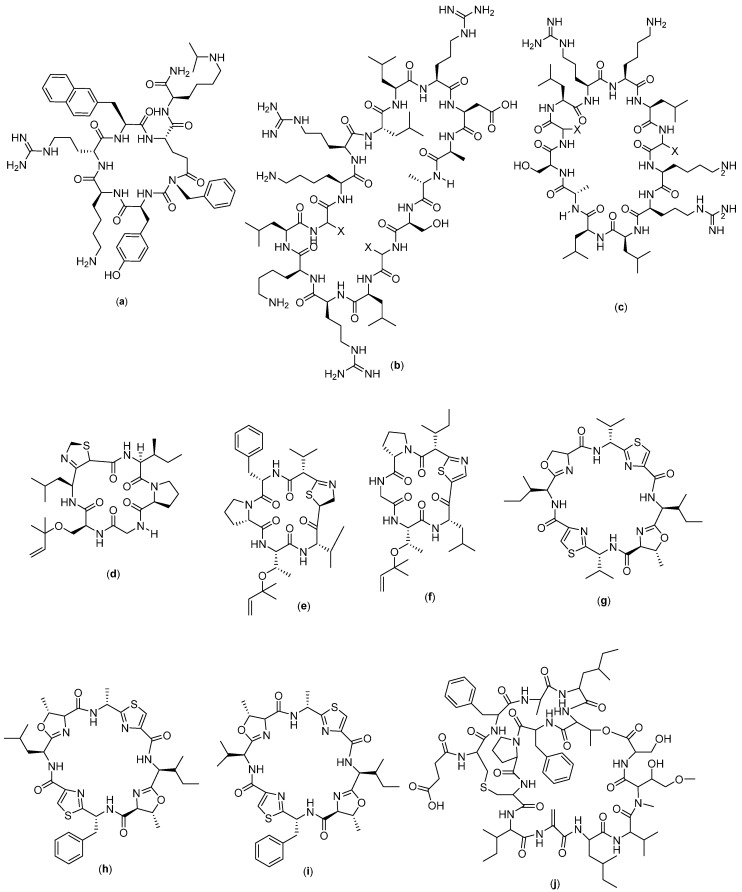
Structure of (**a**) LYS2510924 (**b**) MCOG 1; (**c**) MCOG 2; (**d**) Keenamide; (**e**) Mollamide A; (**f**) Mollamide B; (**g**) Patellamide A; (**h**) Patellamide B; (**i**) Patellamide C; and (**j**) Vitilevuamide.

LY2510924 (Figure 9a) is a cyclic peptide compound with a sequence of Phe-Tyr-Lys-D-Arg--Gly-D-Glu-Lys-NH2 that has been reported to have cytotoxic activity against lymphoma with the mechanism of inhibiting metastasis from tumors. It acts by blocking the migration process and inhibiting the process of cell proliferation by inhibiting signaling on CXCR4. This compound was clinically tested in phase II and the results showed that compound LY2510924 specifically blocked the binding of SDF1 to CXCR4 with an IC_50_ value of 0.079 nM, and inhibited SDF-1-induced GTP binding with a Kb value of 0.38 nM. In human lymphoma U937 cells expressing endogenous CXCR4, LY2510924 inhibited SDF-1-induced cell migration with an IC_50_ value of 0.26 nM [106].

### 4.8. Cyclic Peptide for Blood Cancer

In studies performed by Christensen et al. (2011), stable cyclic peptides were found that were able to internalize into cells and inhibit SET. This compound was obtained by re-engineering MCOTI-II by grafting COG1410 onto the scaffold. This is because COG peptide is a strong antagonist of SET and induces apoptosis in leukemia cancer cell lines. COG peptide binds to SET [107] and increases PP2A activity, which, in turn, inactivates nuclear factor kappa B (NF-κB), a protein complex involved in signaling processes of inflammation and tumor development to facilitate cell penetration [108]. COG peptides have been conjugated to cell-penetrating peptides, which also enhance biological activity, but the poor in vivo stability of these compounds limits their activity. In this study, the optimization of COG1410 compound with MCOTI-II was carried out. This is because cyclotides consist of a head-to-tail cyclic backbone and cystine knots which, together, form the cyclic cystine knot (CCK) motif. The cystine knot is formed by the penetration of one disulfide bond through the macrocyclic ring formed by the other two disulfide bonds and their interconnecting backbone segments. Due to the high stability of the CCK structure, cyclotides are resistant to proteolytic, chemical, and thermal degradation, thus serve as promising templates for the design of new drugs. They have been used as scaffolds for drug design for a variety of conditions, including cancer [109], cardiovascular disease [110], and multiple sclerosis [111], as well as other applications. Cyclotides such as kalata B127 and Momordica cochinchinensis trypsin inhibitors I and II (MCoTI-I/II) are capable of being internalized by mammalian cells and have been classified as: cyclic-cell-penetrating peptides (CCPPs). Previous studies have shown that the uptake of MCoTI-I/II in human cells occurs via endocytosis, and the overall positive charge of the peptide is important for efficient internalization. In a recent study by Camarero and colleagues, MCoTI-I was engineered to combine 12-amino acids into a helical peptide, generating a cyclotide that inhibits the interaction between p53 and the oncogenic protein-binding domains Hdm2 and HdmX, thereby activating the p53 tumor suppressor pathway. This confirms that MCoTI-I can be used as an intracellular delivery vehicle to target protein–protein interactions. The structural stability and cell penetration properties of MCoTI-I/II make it a potentially valuable framework in drug design [112]. From the results of this grafting, two compounds were obtained, namely, MCOG 1 (Figure 9b) and MCOG 2 (Figure 9c), with fairly similar amino acid residues. MCOG 1 has amino acid residues in the form of Ala-Ser-X-Leu-Arg-Lys-Leu-X-Lys-Arg-Leu-Leu-Arg-Asp-Ala, whereas in MCOG 2, they are Ala-Ser-X-Leu-Arg-Lys-Leu-X-Lys-Arg-Leu-Leu. These two compounds have high serum stability and cytotoxic properties in cellular channel CML [113].

Sixth A is a cyclic hexapeptide compound isolated from the notaspidean mollusk *Pleurobranchus forskalii* and has a chemical structure of cyclo-[Gly-Ser (Unk)-Unk-xiIle-Pro]. Keenamide (Figure 9d) is known to have significant cytotoxic properties in several cancer cell lines, which include P-388, A-549, MEL-20, and HT-29. The IC_50_ values for P-388, A-549, and MEL20 were 2.5 µg/mL and 5.0 µg/mL, respectively, for HT-29. However, the mechanism of action of this compound as an anticancer agent is unknown [57].

Mollamide (Figure 9e,f) is a cyclic hexapeptide compound isolated from *Didemnum molle tunicata* in Indonesia. The isolation revealed two mollamide analogues, namely, B and C (3). Mollamide B contains cyclo [Phe-Pro-Thr (Unk) -Unk-Val], whereas mollamide C is cyclo [Gly-Ser (Unk)-Unk-aIle-Pro]. Mollamide B was assayed on several cell lines: H460, MCF7, and SF-268. None of the cell lines displayed an above-average sensitivity to mollamide B. Mollamide C was tested in an in vitro disc diffusion assay, and the differential cell killing among the nine cell types were examined—leukemia (murine L1210 and CCRF-CEM human), five solid tumors (MC38, HCT-116, H125, MCF-7, and LNCaP), and normal murine cells and humans (hematopoietic progenitor cells, CFU-GM). This test is designed to determine large differences in the relative sensitivity of solid, normal cell, and leukemia tumors. The greater the zone of difference in solid tumor colonies and the lower the toxicity for leukemia and normal cells, the higher the selectivity of the compound for solid tumors. Mollamide C has no different differential unit zone against L1210, human colon HCT-116, and human lung H125 (value of 100), and a value of 250 against murine colon 38; hence, it is not considered to be selective towards solid tumors [58].

Patellamide (Figure 9g–i) is a cyclic peptide compound isolated from the ascidian *Lissoclinum patella*. There are three known analogues of patellamide: A, B, and C. The different analogues were tested against murine leukemia cell line P388, and the IC_50_ value recorded was ~5 µg/mL. The exact mechanism of action of these compound analogues as an anticancer agent has not been discovered to date [59].

Vitilevuamide (Figure 9j) is a bicyclic peptide with thirteen amino acids, and it was obtained from the isolation of two marine ascidian species, namely, *Didemnum cuculiferum* and *Polysyncranton lithostrotum*. Vitilevuamide has demonstrated cytotoxicity with an IC_50_ ranging from 6 to 311 nM. In an in vitro test, the compound vitilevuamide was active against p388 lymphocytic leukemia, and it had also increased the lifespan of rats as an animal model with leukemia by 70% at a given dose of 30 g/kg. Vitilevuamide has a positive effect on cell-based assays used for tubulin polymerization inhibitors; thus, the mechanism of action is thought to be due to its inhibition on the tubulin polymer. Vitilevuamide also exhibits non-competitive inhibition of the binding of vinblastine to tubulin. The binding of colchicine to tubulin is stabilized in the presence of vitilevuamide in a manner similar to that of vinblastine. Dolastatin 10 binding is not affected by vitilevuamide at low concentrations, but is inhibited at higher concentrations. GTP binding was also found to be weakly affected by the presence of vitilevuamide [60].

**Figure 10:**
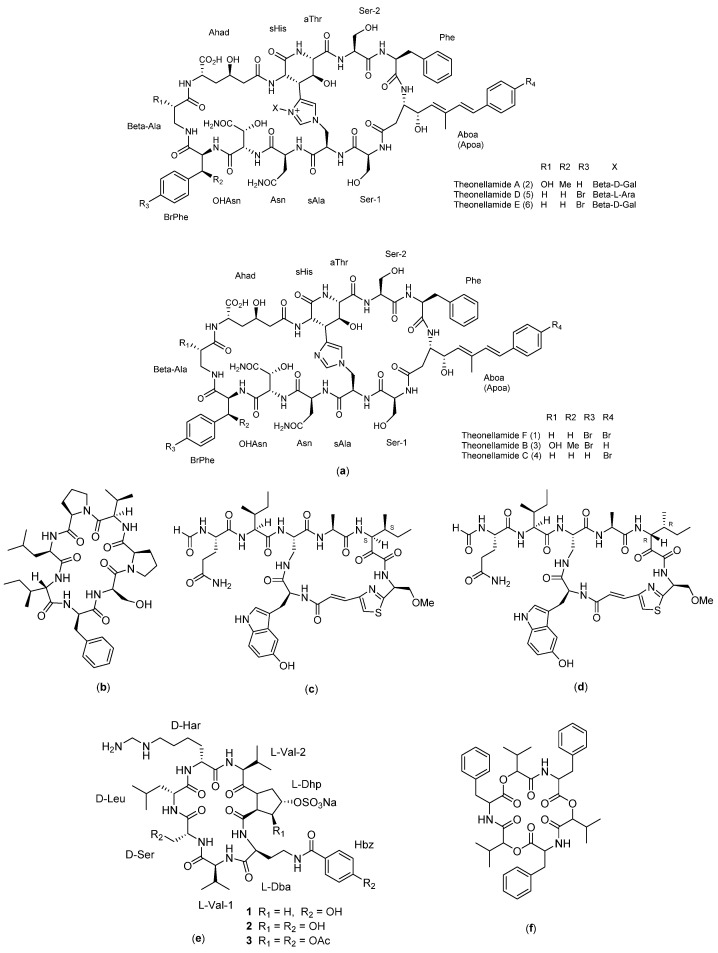
Structure of: (**a**) Theonellamide; (**b**) Stylopeptide 1; (**c**) Calyxamide A; (**d**) Calyzamide B; (**e**) Cupolamide; and (**f**) Beauvericin.

Theonellamide (Figure 10a) is a cyclopeptide compound isolated from the marine sponge *Theonella* sp. Theonellamide A contains D-galactose bound to the free nitrogen of the imidazole, whereas theonellamide D and E have arabinoside and D-galactoside. According to a previously reported study, theonellamides interact with 3β-hydroxysterols in membrane lipids and induce major morphological changes in cultured mammalian cells and yeast by activating Rho1-esis-glucan synthesis [61].

Stylopeptide 1 (Figure 10b) is a cyclopeptide compound obtained from the isolation of sponges in the south and west Pacific Ocean, namely, *Stylotella* sp. and *Phakellia costata*. This compound has a cyclic peptide structure consisting of seven amino acids (Pro-Leu-Ile-Phe-Ser-Pro-Ile). Stylopeptide 1 exhibited strong cancer cell growth inhibition properties against P388 murine lymphocytic leukemia cells, with an ED_50_ value of 0.01 µg/mL. This compound has not been explored in depth regarding its mechanism as an anticancer agent [27].

Calyxamide (Figure 10c,d) is a cyclopeptide compound containing 5-hydroxytryptophan and thiazole groups isolated from a marine sponge in Japan, the lithistid sponge *D. calyx*, which is a source of diversity in natural products and has a unique structural and activity. The structure is similar to that of the F, G, H, J, and K ramamides isolated from the marine sponge Theonella sp. located in Okinawa, Japan. Calyxamides A and B showed moderate cytotoxicity against murine P388 leukemic cells, with IC_50_ values of 3.9 and 0.9 µM, but have not been explored further regarding their mechanism of action [62].

Cupolamide A (Figure 10e) is a cyclic peptide compound composed of seven amino acids. This compound is known to exhibit activity against murine P388 leukemia cells with an IC_50_ value of 7.5 µM, but it is not cytotoxic to thrombin. The structure of the compound cupolamide A is similar to that of gamma-aminobutyric acid (GABA), which may explain its potential activity on the central nervous system; however, this requires further study. The structure of cupolamide B consists of L-Dba, L-Val (x2), D-Ser, D-Leu, D-Har, and L-3S, 4SDhp, whereas the cupolamide A cyclic sequence consists of Val-Dba-HypVal-Har-Leu-Ser-Val [63,64].

Beauvericin (BEA) (Figure 10f) is a compound isolated from the fungus *Beauveria bassiana* and various species of *Fusarium mushrooms.* BEA is a hexadecapeptide with a structure of cyclo [D-OVal-N (Me) Phe-D-OVal-N (Me) Phe-D-OVal-N (Me) Phe] [65]. It has the ability to induce cell death in leukemic cells (CCRF-CEM) in humans through the apoptotic pathway. In a previously conducted study, CCRF-CEM cells were treated with BEA at various concentrations between 1 and 10 µM for 24 h. The data revealed that BEA-induced cell death in CCRF-CEM cells exhibited aggravation with dose and time. Increased cytosolic caspase-3 activity was also observed in BEA-treated cells in a dose-dependent manner. Thus, the currently available data suggest that the cytotoxicity of BEA happens through activation of the apoptotic pathway by releasing cytochrome c from the mitochondria, leading to increased caspase-3 activity, and ultimately leading to cell killing [65].

### 4.9. Cyclic Peptide for Myeloma

**Figure 11:**
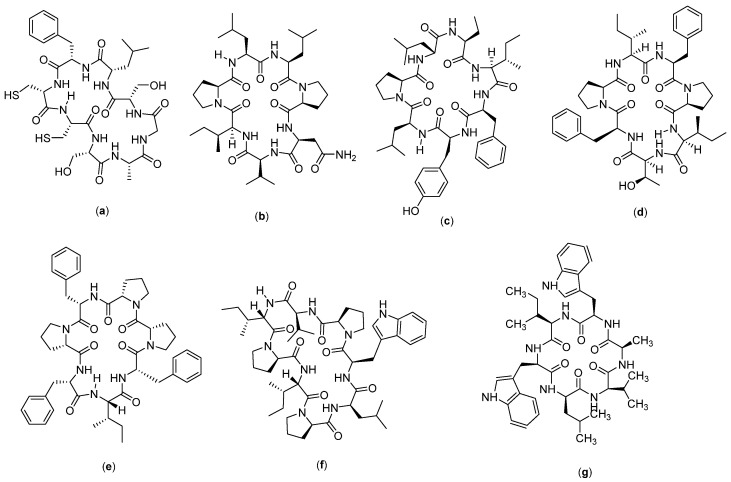
Structure of: (**a**) ALOS4; (**b**) Reniochalistatin A; (**c**) Reniochalistatin B; (**d**) Reniochalistatin C; (**e**) Reniochalistatin D; (**f**) Reniochalistatin E; and (**g**) Nocardiotide A.

ALOS4 (Figure 11a) is a cyclic peptide compound which has nine amino acids in the form of (Cys-Ser-Ser-Ala-Gly-Ser-Leu-Phe-Cys)-OH. This compound was designed from the phage library search, and the selection of the α_v_β_3_ integrin, as well as the potential activity of this compound against melanoma cancer, was evaluated in a human xenograft model (A375). It was found that the compound reduces the mass and volume of melanoma tumors, but on tissue examination, it did not show significant effects on the proliferation, mitotic index, tissue vascularization, or reduction in tumor markers [114]. This compound is thought to have a role in integrin-mediated pathways, through which it exerts its anticancer effects by modulating immune responses. Integrins themselves are known to have important roles in progression [115], metastasis [116], invasion [117], and angiogenesis [118]. Structurally, the α_v_β_3_ integrin has a common integrin binding motif and recognition of RGD with the Arg-Gly-Asp sequence along with several extracellular matrix (ECM) proteins, including vitronectin, fibronectin, and fibrinogen. Due to its high expression in the active and angiogenetic proliferation of endothelial cells, α_v_β_3_ integrin has become a target for cancer therapy [119] and is considered a cancer prognostic biomarker [120], which correlates well with tumor progression and invasion in cancers such as gliomas [121], prostate carcinoma [122], osteosarcoma [116], breast cancer [120], and melanoma [123]. The α_v_β_3_ integrin in transformed melanocytes mediates tumor angiogenesis and is associated with the organ-specific metastasis of human malignant melanoma. Several possible therapeutic approaches have been suggested to target α_v_β_3_ integrin using monoclonal antibodies and cyclic peptide RGD antagonists. These agents could function by preventing tumor growth, angiogenesis, and metastasis by inhibiting the activity of α_v_β_3_ integrin [114].

Reniochalistatin (Figure 11b–f) is a cyclic peptide compound isolated from the marine sponge *Reniochalina stalagmitis* obtained from Yongxing Island in the South China Sea. There are five types of reniochalistatin compounds, namely, A–E. Reniochalistatin A has the chemical structure of cyclo(Pro-Asn-Val-Ile-Pro-Leu-Leu), reniochalistatin B consists of cyclo(Pro-Ile-Phe-Tyr-Leu-Pro-Leu-Pro-Leu), reniochalistatin C consists of cyclo(Pro-Ile-Phe-Pro-Ile-Tyr-Phe), reniochalistatin D is formed by cyclo(Pro-Phe-Pro-Phe-Ile-Phe-Pro), and reniochalistatin E consists of cyclo(Pro-Leu-Trp-Pro-Val-Ile-Pro-Ile). The five compounds were tested on several cancer cell lines (RPMI-8226, MGC-803, HL-60, HepG2, and HeLa). The obtained data showed that the compound reniochalistatin E exerts cytotoxicity against RPMI-8226 and gastric myeloma cells MGC-803, with IC_50_ values of 4.9 and 9.7 µM, respectively. No activity was recorded against HL-60 leukemia and HepG2 hepatoma (IC_50_ > 20 µM), and low levels of activity were observed on cervical HeLa cells (IC_50_ 17.3 µM) [66].

Nocardiotide A (Figure 11g) is a cyclic hexapeptide compound isolated from marine biota, called *Nocardiopsis* sp. Nocardiotide is formed by six amino acids (Trp-Ile-Trp-Ala-Val-Leu). It is known to have cytotoxicity on the multiple myeloma cell line MM.1S, human HeLa cervical carcinoma, and murine CT26 colon carcinoma cells, with IC_50_ values of 8, 11, and 12 µg/mL, respectively [124]. This compound has also been successfully synthesized by the peptide synthesis method through combinations between solid and liquid phase peptide synthesis techniques, with a yield of 16%. However, no anticancer activity was recorded on the cell lines tested, based on the low IC_50_ values obtained [67] (Table 2).

## 5. Conclusions: Future and Prospect

There are many types of cyclic peptide compounds that have been shown to have potential cytotoxicity against numerous cancers based on the currently available data. In this review, the authors have summarized and compared the different cyclic peptide compounds. The potency in breast cancer cell lines follows the sequence AFPep > coibamide > RA-V > bacillistatin > sansalvamide A > schleritodermin A > wewakazole > cyclosaplin > galaxamide > cyclo-CLLFVY > cyclo-SGWTVVRMY. In lung cancer, the potency is reported as urukthapelstatin > aurilide > scleritodermin A > axinellin > psammosilenin B. In colon cancer, nannocystin > Dol-10 cyclic > RA-XII > C25 > theopapuamide > stereocalpin > sansalvamide G > longicalcynin. In prostate cancer, IB01212 > kahalalide F > lagunamides, whereas in liver cancer, stylissatin > dianthin > leucamide > grifficyclocin B > phakillestatin > clavustide. In blood cancer, the compounds showed potency in the order of vitilevuamide > stylopeptide > calyxamide > theonellamide > pattelamide > canceride > cupolamide > beauvericin > MCOT > mollamide, and in myeloma, the order was raniochalistatin > nocardiotide > ALOS4. Each compound has their own mechanism of action, which may also act synergistically with current available chemotherapeutics. Nevertheless, more in-depth studies are necessary before they can be developed as anticancer agents to establish their safety and efficacy, in addition to improving the selectivity of the peptides towards cancer cells in vivo. However, there are several challenges in the development of cyclic peptide as anticancer agents, such as the availability and recovery of cyclic peptide compounds from natural resources that could be limited in amount. This calls for other production methods such as chemical synthesis. However, the complexity in the cyclic peptides’ structure may pose another challenge. Compounds such as galaxamide, bacillistatin, sansalvamide, cyclo-CLLFVY, longicalcynin, grifficyclocin B, dianthin, phakellistatin, leucamide, stylissatin, clavatustide, keenamide, mollamide, stylopeptide, beauvericin, reniochalistatin, and nocardiotide may be synthesized by the general peptide synthesis method, but the overall yield may be insufficient. Therefore, a more innovative solution may be needed to ensure success in the development of cyclic peptide compounds for clinical application. Computational analysis will also be needed to understand the interactions between cyclic peptides and the targets. The currently available data summarized in this review will be useful for driving this initiative forward, with the hope that cyclic peptides may be used in clinical practice in the near future.

## Figures and Tables

**Table 1:** Isolation of cyclic peptides from natural sources.

Compound	Source	Natural Source	Ref.
RA-V	Plant	*Rubia cardifolia* L.	[24]
Apratoxin A	Bacteria	*Moorea producens and Moorea bouillonii*	[30,31]
Coibamide A	Bacteria	*Leptolyngbya* sp.	[32]
Galaxamide	Algae	*Galaxaura filamentosa*	[33]
Sansalvamide A	Fungi	*Fusarium*	[25]
Cyclosaplin	Plant	*Santalum album* L.	[34]
Wewakazole	Bacteria	*Lyngbya majuscula and Moorea producens*	[26]
Scleritodermin A	Sponge	*Scleritoderma nodosum*	[35]
Psammosilenin B	Plant	*Psammosilene tunicoides*	[36]
Lyngbyabellin	Bacteria	*Lyngbya majuscula*	[37]
Aurilide	Bacteria	*Dolabella auricularia and Lyngbya majuscule*	[38]
Urukthapelstatin	Bacteria	*Thermoactinomycetaceae Mechercharimyces asporophorigenens*	[39]
Axinellin	Sponge	*Axinella carteri*	[40]
RA-XII	Plant	*Rubia yunnanensis Diels*	[41]
Longicalcynin A	Plant	*Dianthus superbus*	[42]
Sansalvamide G	Fungi	*Fusarium*	[43]
Nannocystin	Bacteria	*Nannocystis* sp.	[44]
Theopapuamide	Sponge	*Siliquariaspongia mirabilis*	[45]
Stereocalpin	Sponge	*Ramalina terebrata*	[46]
Dollastatin 10	Mollusca	*Dolabella auricularia*	[47]
Kahalalide F	Algae	*Bryopsis pennata*	[48]
IB-01212	Fungi	*Clonostachys* sp.	[49]
Lagunamides	Bacteria	*Lyngbya majuscula*	[50]
Scopularides	Fungi	*Scopulariopsis brevicaulis,*	[51]
Grifficyclocin B	Plant	*Goniothalamus*	[14]
Dianthin	Plant	*Dianthus superbus*	[52]
Phakillestatin 6	Sponge	*Chuuk archipelago*	[53]
Leucamide	Sponge	*Leucetta mikrorapis*	[54]
Stylissatin	Sponge	*Stylissa masse*	[55]
Clavatustide	Fungi	*Aspergillus clavatus*	[56]
Keenamide	Mollusca	*Pleurobranchus forskalii*	[57]
Mollamide	Sponge	*Didemnum molle tunicata*	[58]
Patellamide	Bacteria	*Lissoclinum patella.*	[59]
Vitilevuamide	Ascidian	*Didemnum cuculiferum and Polysyncranton lithostrotum.*	[60]
Theonellamide	Sponge	*Theonella* sp.	[61]
Stylopeptide 1	Sponge	*Stylotella* sp. *and Phakellia costata*	[27]
Calyxamide	Sponge	*D. calyx*	[62]
Cupolamide A	Sponge	*Theonella cupola*	[63,64]
Beauvericin	Fungi	*Beauveria bassiana*	[65]
Reniochalistatin	Sponge	*Reniochalina stalagmitis*	[66]
Nocardiotide A	Sponge	*Nocardiopsis* sp.	[67]

**Table 2:** Cyclic peptides as anticancer agents and their mechanisms of action.

Cyclic Peptide as Anticancer Agents			
Compound	Types of Cancer	Mode of Action	Ref.
RA-V	Breast cancer	Triggers apoptosis in the mitochondrial pathway	[24]
RA-XII	Colon cancer	RA-XII suppresses early stages of autophagy by activating the Akt–mTOR pathway and inhibiting autophagosome formation	[41]
Cobamide A	Breast cancer	Inhibits EGFR expression and promotes EGFR degradation through a proteasome-dependent pathway	[71]
Apratoxin A	Breast cancer	Interferes with cellular proteostasis and induces both of them to form non-apoptotic cell death	[72]
Galaxamide	Breast cancer	Induces apoptosis by disrupting the potential of mitochondrial membranes, producing ROS, and activating oxidative-stress-mediated signaling in breast cancer cells	[28]
Bacillistain	Breast cancer	-	[29]
Cyclosaplin	Breast cancer	Sensitizes MDA-MB-231 cells by possibly binding to EGFR and inducing apoptosis	[34]
Sansalvamide A	Breast cancer	Inhibits a key protein involved in tumor development (Hsp90)	[77]
Cyclo-CLLFVY	Breast cancer	Inhibits the dimerization and transcriptional activity of HIF-1 by binding to the PAS-B domain of HIF-1α, reducing the signaling of hypoxia-mediated responses for HIF-1 in various cell lines, without the closely related influence of HIF function	[81]
Cyclo-SGWTVVRMY	Breast cancer	Inhibitors identified significantly reduce mitosis, proliferation, and colony formation potential, although the compound does not affect mitosis in cells with lower glycolytic fluxes	[82]
AFPep	Breast cancer	Has the ability to stop the growth of human breast cancer xenografts grown in SCID mice	[85]
Wewakazole	Breast cancer	-	[26]
Scleritodermin A	Breast cancer	Inhibits the tubulin polymer, causing microtubule disruption	[35]
Psammosilenin	Lung cancer	-	[36]
Lyngbyabellin	Lung cancer	Induces apoptosis through the impaired cytokinesis of cellular microfilament networks	[37]
Aurilide	Lung cancer	Inhibits mitochondrial fragmentation and apoptosis	[38]
Urukthapelstatin A	Lung cancer	-	[125]
Axinellin	Lung cancer	-	[40]
Longycalcynin	Colon cancer	Impairs the integrity of the lysosomal membrane	[93]
Sansalvamide G	Colon cancer	Induces the apoptosis of B16 cells	[95]
Nannocystin	Colon cancer	Targets eukaryotic translation elongation factor 1 A (eEF1A)	[97]
C25	Colon cancer	Significantly activates CD8+ T cells. An increase in CD8+ T will result in a significant decrease in FOXP3+ at the tumor site; thus, it can be used as an alternative for immunotherapy in cancer	[98]
Theopapuamide	Colon cancer	-	[45]
Stereocalpin	Colon cancer	-	[46]
Cyclo Dol-10	Colon cancer	Reduces apoptosis in cancer cells	[47]
Kahalide F	Prostate cancer	Induces action mostly by necrotizing cell processes involving oncosis rather than apoptosis	[102]
IB-01212	Prostate cancer	-	[49]
Lagunamide	Prostate cancer	-	[50]
Scopularides	Pancreatic cancer	-	[51]
Grifficyclocin	Liver cancer	Induces apoptosis and the arrest of G2/M HCC cells; activation of the caspase pathway may be involved	[14]
Dianthin E	Liver cancer	-	[103]
Phakellistin 6	Liver cancer	-	[53]
Leucamide	Liver cancer	-	[54]
Stylissatin	Liver cancer	Inhibits the production of nitric oxide in murine macrophage cells	[55]
Clavatustide	Liver cancer	Regulates the G1-S transition in liver cancer cell lines	[56]
LY2510924	Lymphoma cancer	Inhibits metastasis from tumors by blocking the migration process and inhibiting the process of cell proliferation by inhibiting signaling on CXCR4 or a strong and selective antagonist of CXCR4	[106]
MCOG 1 and 2	Blood cancer	Antagonist of SET and induces apoptosis	[113]
Keenamide	Blood cancer	-	[57]
Mollamide	Blood cancer	-	[58]
Patellamide	Blood cancer	-	[59]
Vitilevuamide	Blood cancer	Inhibits the tubulin polymer	[60]
Theonellamide	Blood cancer	Interacts with 3β-hydroxysterols in membrane lipids and induces major morphological changes in cultured mammalian cells and yeast by activating Rho1-esis-glucan synthesis	[61]
Stylopeptide	Blood cancer	-	[27]
Calyxamide	Blood cancer	-	[62]
Cupolamide	Blood cancer	-	[63,64]
Beauvericin	Blood cancer	Activates the apoptotic pathway by releasing cytochrome c from the mitochondria, leading to increased caspase-3 activity, ultimately resulting in the morphology characteristics of the apoptotic markers	[65]
ALOS4	Myeloma cancer	Inhibits integrin signal transduction, tumor growth, angiogenesis, and metastasis, blocking αvβ3 with monoclonal antibodies, cyclic peptide RGD antagonists, or other antagonists	[114]
Reniochalistatin	Myeloma cancer	-	[66]
Nocardiotide	Myeloma cancer	-	[124]
